# Gut Microbiota and Bone Diseases: A Growing Partnership

**DOI:** 10.3389/fmicb.2022.877776

**Published:** 2022-05-06

**Authors:** Yu Chen, Xin Wang, Chunlei Zhang, Zhiyong Liu, Chao Li, Zhigang Ren

**Affiliations:** ^1^Gene Hospital of Henan Province, Precision Medicine Center, The First Affiliated Hospital of Zhengzhou University, Zhengzhou, China; ^2^Academy of Medical Sciences, Zhengzhou University, Zhengzhou, China; ^3^Department of Orthopaedic Surgery, The Affiliated Cancer Hospital of Zhengzhou University, Zhengzhou, China; ^4^Bone Tumour and Bone Disease Department II, Zhengzhou Orthopaedic Hospital, Zhengzhou, China; ^5^Department of Infectious Diseases, The First Affiliated Hospital of Zhengzhou University, Zhengzhou, China

**Keywords:** gut microbiota, bone diseases, osteoporosis, osteoarthritis, rheumatoid arthritis, bone tumor

## Abstract

Gut microbiota is key to human health and disease. Convincing studies have demonstrated that dysbiosis in the commensal gut microbiota is associated with intestinal and extra-intestinal diseases. Recent explorations have significantly contributed to the understanding of the relationship between gut microbiota and bone diseases (osteoporosis, osteoarthritis, rheumatoid arthritis, and bone cancer). Gut microbiota and its metabolites may become associated with the development and progression of bone disorders owing to their critical role in nutrient absorption, immunomodulation, and the gut–brain–bone axis (regulation hormones). In this work, we review the recent developments addressing the effect of gut microbiota modulation on skeletal diseases and explore a feasible preventive approach and therapy for bone diseases.

## Introduction

The microorganisms in the human gastrointestinal tract, henceforth referred to as gut microbiota (gut microbiota; most of them bacteria but also viruses, fungi, archaea, and protozoa), have a symbiotic relationship with the human body (Bäckhed et al., 2005). Bacteria are an abundant component of the microbiome, particularly in the gut, where the ratio of gut bacteria to the host cells is much closer to 1:1 (Sender et al., 2016). However, gut microbiota encodes more than 3 million genes, approximately 150 times the number of genes in the host genome; it regulates various functions of the host and consequently influences host health, phenotype, and diseases. Thus, the gut microbiome is now considered as a virtual metabolic organ in the host body (Eckburg et al., 2005; Rath and Dorrestein, 2012; Vyas and Ranganathan, 2012). In recent years, different levels of evidence have demonstrated the pivotal role of gut microbiota in both human health and diseases. Gut microbiota participates in the regulation of various physiological processes in the human body, including modulation of host physiological development and functions, such as nutrient absorption and production, immune function, metabolic balance, and resistance to pathogens at different period of ages (Dominguez-Bello et al., 2019). It is also associated with many complex human intestinal and extra-intestinal diseases, such as inflammatory bowel disease, obesity, diabetes, cardiovascular disease, cancer, and depression (Guarner and Malagelada, 2003; Wang et al., 2011; Valdes et al., 2018; Sepich-Poore et al., 2021). Furthermore, gut microbiota strongly interacts with certain drugs to influence their response and efficacy (Vich Vila et al., 2020).

Bone disorders, such as osteoporosis (OP), osteoarthritis (OA), and rheumatoid arthritis (RA), present one of the major threats to human health with the aging of society (Briggs et al., 2016). The most common characteristics of these diseases are pain, reduced physical function, significant disability, and increased mortality (Xia et al., 2019). The prevention and treatment of most skeletal disorders include pharmacological interventions (e.g., non-disease-specific drugs that ameliorate pain or more specific ones targeting the pathophysiology of skeletal diseases) and non-pharmacological interventions (e.g., an active lifestyle; an adequate intake of dietary protein, calcium, and vitamin D; and weight management; Papageorgiou and Biver, 2021). Despite the continuous development of these treatments, which indeed relieve the primary pain and improve the quality of life of patients, there is still a considerable proportion of patients gain little or no benefit and even experience adverse effects (Tu et al., 2018). For example, Opioids, as the potent analgesics for their efficacy in ameliorating pain in chronic bone disorders, are concerning due to associated addiction, gastrointestinal symptoms, and respiratory inhibition in a substantial proportion of patients (Wang and Roy, 2017). Therefore, this global challenge requires urgent and feasible solutions to improve the outcomes of these diseases.

**Figure 1:**
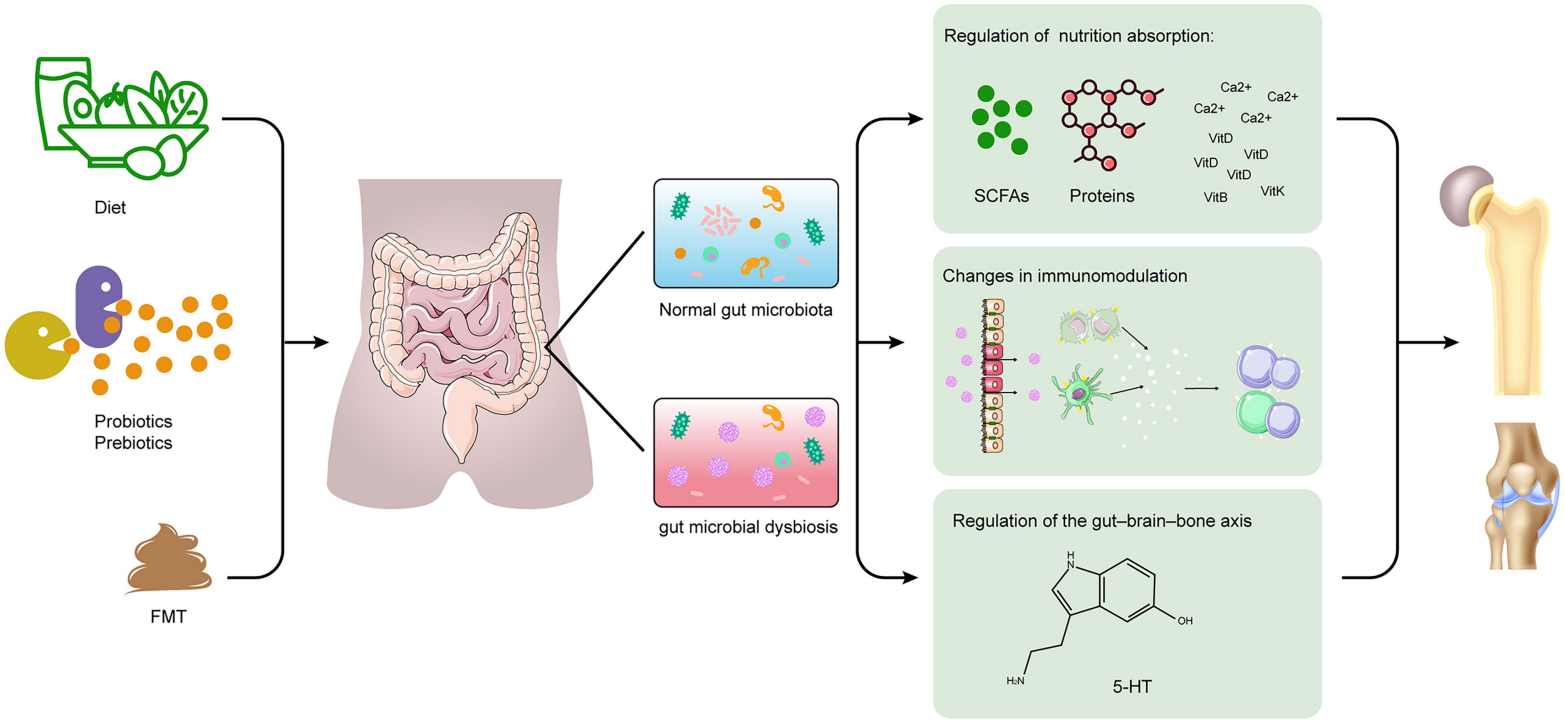
Schematic representation for the role of gut microbiota in bone health and disease. The potential mechanisms include (1) changes in nutrition absorption (i.e., increase in microbial metabolites with health benefits, such as SCFAs); (2) changes in immunomodulation (i.e., regulation of immune cells and cytokines); (3) regulation of the gut–brain–bone axis (i.e., 5-HT). FMT, fecal microbiota transplantation; SCFAs, short-chain fatty acids; Ca, Calcium; VitD, vitamin D; VitB, vitamin B; VitK, vitamin K; and 5-HT, 5-hydroxytryptamine.

There has been growing interest in the role of gut microbiota as an inducer or modulator of bone health and diseases, which is predominantly driven by an improved understanding of the crucial role of gut microbiota and its metabolites in maintaining structural and functional homeostasis (Zmora et al., 2019). The studies with different levels of evidence supporting these associations range from those in animal models to human studies. In the present review, we summarize the communication between gut microbiota and the host that contributes to the development and progression of some bone diseases and discuss potential mechanisms underlying these diseases. We have classified the potential mechanisms that link gut microbiota to bone health into three categories: (1) nutrient absorption, (2) immunomodulation, and (3) gut–brain–bone axis (Figure 1). Furthermore, we describe in detail the relationship of gut microbiota with skeletal outcomes, such as OP, OA, and RA and its role in bone tumor.

## Role of Gut Microbiota in Nutrient Absorption

Gut microbiota provides the host with essential capacities for the fermentation of non-digestible substrates, such as dietary fiber and endogenous intestinal mucus, production of various vitamins, biotransformation of bile acids, and synthesis of essential and non-essential amino acids. Notably, these functionalities have beneficial or detrimental effects on bone through alterations in gut microbiota composition and function.

### Calcium

Calcium is a key nutrient necessary for human bone health, and its deficiency may precipitate or exacerbate osteopenia and OP (Holick, 2007). It has been found that increased dietary calcium intake improves bone mineral density in a short time. Calcium intake is a significant predictor of the total body bone mass (Zhu et al., 2008). It was shown that probiotic supplement (*Lactobacillus* strains) is beneficial for calcium transport and uptake in mice (Raveschot et al., 2020). Probiotics, which are defined as “live microorganisms” confer health benefits to the host when administered in adequate amounts (Morelli and Capurso, 2012). In line with this, a human trial demonstrated that supplementation with *Lactobacillus helveticus* fermented milk in postmenopausal women increased serum calcium levels and reduced serum parathyroid hormone (PTH) levels (Narva et al., 2004). The changes in calcium and PTH suggest a benefit to bone health.

In addition, studies on prebiotics provide several indications for the regulation of calcium absorption by the manipulation of gut microbiota. Prebiotics cannot be hydrolyzed and absorbed by the gut; however, they are defined as substrates that can be selectively used by host gastrointestinal micro-organisms resulting in a health benefit (Bindels et al., 2015). All compounds considered prebiotics are microbiota accessible carbohydrates or fermentable dietary fiber, however, the reverse is not true (Deehan et al., 2017). Prebiotics, especially non-digestible oligosaccharides are the most promising and the most investigated substances in association with calcium intake and metabolism (Scholz-Ahrens et al., 2007). In an experiment with male Sprague Dawley rats, the consumption of galactooligosaccharides (GOS) led to increased calcium and magnesium absorption and bone density (Weaver et al., 2011). Moreover, in growing Sprague Dawley rats, calcium and magnesium absorption increased in a dose–response manner with GOS supplementation (Weaver et al., 2011). These benefits to mineral utilization were associated with decreased cecal pH, increased cecal wall and content weight, and increased proportion of *Bifidobacterium* (Weaver et al., 2011). Another study found that calcium absorption in postmenopausal women was higher in the 20 g GOS treatment group than in the control group (van den Heuvel et al., 2000).

Probiotics and prebiotics improve calcium bioavailability through several potential mechanisms. First, the ingestion of probiotics and prebiotics is associated with an increase in cell density, intestinal crypt depth, and blood flow, which may increase intestinal surface area and enhance calcium absorption (Narva et al., 2004; Raschka and Daniel, 2005). Second, the transformation of prebiotics to short-chain fatty acids (SCFAs) under metabolism by gut microbiota lower the pH in the intestinal lumen; such changes may hinder the formation of calcium phytate/oxalate complexes and increase calcium solubility, thereby increasing the amount of available calcium for absorption (Weaver, 2015). Third, probiotics and prebiotics may upregulate the expression of calcium transporters, which increases calcium absorption, reduces PTH, and has downstream effects on bone by reduction in bone resorption (Ohta et al., 1998; Raveschot et al., 2020). The potential beneficial effect of calcium ingestion on skeletal health may be associated with the modifications of intestinal microbiota and integrity. Calcium supplementation or high calcium diet in animal models has been demonstrated to increase the number of potentially beneficial bacteria (e.g., *Lactobacillus*, *Ruminococcaceae*, and *Akkermansia*) and SCFA production, maintain intestinal integrity, prevent endotoxemia, and regulate tight junction proteins (Hwang et al., 2013; Gomes et al., 2015; Chaplin et al., 2016; Nadeem Aslam et al., 2016; Cha et al., 2020).

### Vitamin D

It is well established that vitamin D has an immense influence on promoting gut calcium and phosphorus absorption and bone metabolism (Holick, 2007). The gut microbiome regulates vitamin D metabolism and function, as evidenced by the finding that germ-free (GF) mice have defective vitamin D metabolism (low 1, 25-dihydroxvitamin D levels; hypocalcemia), whereas colonization of GF mice with microbiota showed recovered levels of 1, 25-dihydroxvitamin D and calcium (Bora et al., 2018). Furthermore, a clinical trial showed that the oral probiotic supplementation of *Lactobacillus reuteri* NCIMB 30242 increases the level of mean circulating 25-hydroxyvitamin D (Jones et al., 2013). The recent discovery that the vitamin D receptor (VDR) is highly expressed in the gastrointestinal tract suggests that the effects of vitamin D are exerted *via* the endocrine and immune systems. Vitamin D activates the VDR and plays a role in maintaining the intestinal epithelial barrier function and gut microbiota eubiosis (Barbáchano et al., 2017). Dysbiotic gut microbiota profiles with an increased abundance of the phyla *Bacteroidetes* and *Proteobacteria* and decreased abundance of the phyla *Firmicutes* and *Deferribacteres* in the feces were reported in VDR knockout mice vs. wild-type mice, and the disorder was more likely to induce colitis in VDR knockout mice, whereas after vitamin D supplementation, inflammation was controlled and the proportion of *Firmicutes* and *Deferribacteres* was restored (Ooi et al., 2013). Additional investigations provided evidence that vitamin D deficiency induces gut inflammation by lower expression of E-cadherin at epithelial surfaces and immune cells, and fewer tolerogenic dendritic cells in mouse models (Ooi et al., 2013). Such changes (increased host inflammation) may allow pathogens to outcompete commensal bacteria, resulting in the upregulation or downregulation of immune responses (Ooi et al., 2013). The finding is consistent with observations in human studies, which showed that the differences in gut microbiota composition and inflammatory markers are in accord with vitamin D intake or vitamin D levels (Charoenngam et al., 2020). Moreover, multiple epidemiological studies have shown that humans with low serum vitamin D levels are at increased risk of multiple adverse health outcomes including OP, cancer, autoimmune diseases, and cardiovascular disease (Bikle, 2016). Therefore, vitamin D supplementation may reinstate a healthier gut microbiome profile and weaken inflammation and it is important to ensure sufficient vitamin intake in daily life.

### Dietary Fiber

Dietary fiber (defined as edible carbohydrate polymers or complex carbohydrates) is resistant to the endogenous digestive enzymes and thus is neither hydrolyzed nor absorbed in the small intestine (Jones, 2014) because the human genome encodes a limited number of glycoside hydrolases and no polysaccharide lyases (collectively referred to carbohydrate-active enzymes or CAZymes; Cantarel et al., 2012). In contrast, the gut microbiome is estimated to encode thousands of CAZymes (Cantarel et al., 2012). Thus, the degradation and fermentation of dietary fiber are one of the dominant functions of gut microbiota (multiple common genera including *Phascolarcto bacterium*, *Roseburia*, *Bacteroides*, *Clostridium*, *Ruminococcus*; Labbé et al., 2014). Carbohydrate metabolism is a major source of energy for gut epithelial cells and a minor supply of nutrients and energy source for gut microbe growth and proliferation (such as *Desulfotomaculum* spp.; Guarner and Malagelada, 2003). Moreover, it produces SCFAs and hydrogen gas (Wong et al., 2006).

Studies have investigated the association between dietary fiber and skeletal outcomes involving SCFAs, such as signaling molecules regulating bone homeostasis (bone formation and resorption) either by inhibiting histone deacetylases (HDACs) or by acting as ligands for several G protein-coupled receptors. They thus affect various physiological processes and may contribute to bone health and disease. Butyrate, the most abundant SCFA, promotes osteoblast differentiation by inhibiting HDACs (Lee et al., 2006) and stimulates mineralized nodule formation and osteoprotegerin (OPG) expression (Katono et al., 2008) to promote bone formation. It also directly stimulates osteoblast activation and bone formation *via* T regulatory cell-mediated regulation of WNT10b expression in CD8^+^ T cells by producing TGFβ (Tyagi et al., 2018). SCFAs other than butyrate, also have direct effects on osteoclasts (Rahman et al., 2003; Katono et al., 2008). They directly induce metabolic reprogramming of osteoclast precursors, resulting in enhanced glycolysis at the expense of oxidative phosphorylation, thereby downregulating essential osteoclast genes, such as *TRAF6* and *NFATc1*, and this metabolic process does not require the participation of G protein-coupled receptors (Lucas et al., 2018). Moreover, the indirect effects of SCFAs on osteoclasts may contribute to their ability to induce T regulatory (Treg) cells, which have been shown to suppress osteoclast differentiation (Zaiss et al., 2007, 2010). In summary, these findings suggest that SCFAs are potent regulators of bone metabolism and homeostasis. However, excessive SCFA intake has adverse effects on the host. A high-fiber diet in mice, which causes a significant increase in butyrate in the gut, is associated with a markedly increased risk of susceptibility to pathogenic *Escherichia coli* O157:H7; it is likely that the intestinal tissue of the mice with a high-fiber diet bound to more Shiga toxin 1 and expressed more globotriaosylceramide (Zumbrun et al., 2013).

Gut microbial enzymes also have been directly implicated in the conversion of other specific products; for example, insulin-like growth factor 1 (IGF-1), produced predominantly in the liver in response to food intake and regulated by microbes and microbial products, was the first metabolite identified to be a mediator in the gut–bone axis (Yan et al., 2016; Novince et al., 2017).

Nutrient absorption is affected by host diet, and the dietary ingredients have an important effect on host health (Valdes et al., 2018). Adequate consumption of dietary protein is important for maintaining bone health because proper dietary intake causes positive changes in gut microbiota and promotes the absorption of nutrients by the intestinal epithelial mucosal barrier, which is beneficial for skeletal metabolism (Rizzoli et al., 2018). In turn, the gut microbiome is implicated in protein metabolism and utilization. For example, the intestinal bacteria hydrolyze undigested proteins into amino acids and synthesize essential amino acids from nitrogen sources (Davila et al., 2013; Neis et al., 2015). Intestinal microbes also play a key role in the synthesis of vitamin B and K and bile acid metabolism (LeBlanc et al., 2013; Guzior and Quinn, 2021). Vitamin B and K are essential for bone health (Boskey et al., 1998; Hoang et al., 2003; Poundarik et al., 2012; Stone et al., 2017), and bile acids may play a key role in the regulation of calcium absorption (Long et al., 2017). Therefore, in addition to gut microbiota homeostasis, a balanced diet and an appropriate ratio of dietary fiber, proteins, and minerals positively affect the bone health. Otherwise, it may negatively affect the bone health, leading to skeletal diseases.

## Role of Gut Microbiota in Immunomodulation

**Figure 2:**
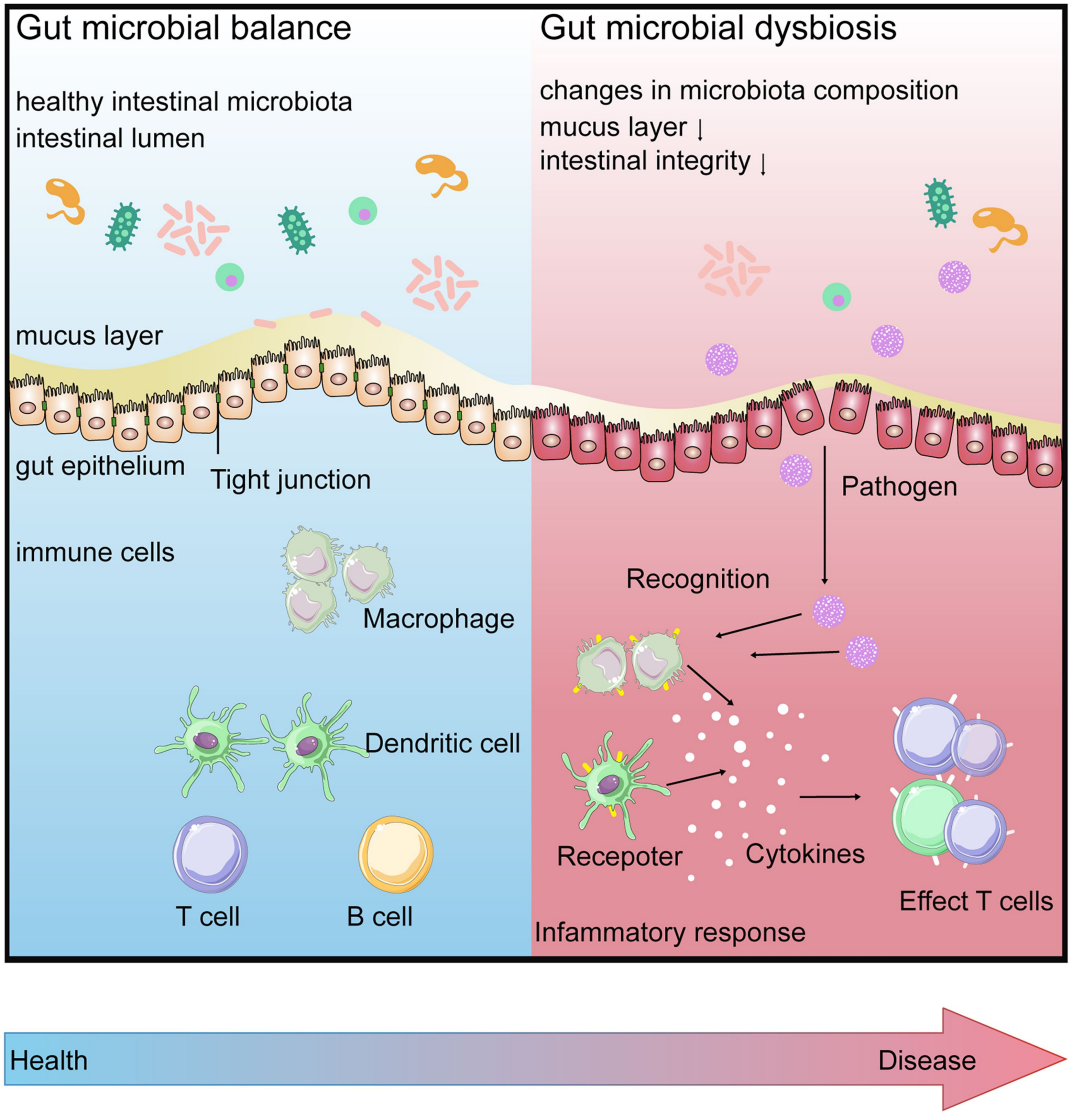
Gut microbial balance (BLUE) vs. gut microbial dysbiosis (RED). When in intestinal homeostasis, gut barrier contributes to surveillance the gut for the entrance of any pathogens. Disruption of the homeostatic relationship between the gut microbiota and gut barrier, the increasing intestinal permeability is involved in further increasing inflammation *via* gut mucosal immune system, leading to disease.

Interactions between gut microbiota and the host immune system begin at birth. In the gastrointestinal tract, numerous microbial communities present on the mucosal surface or in the intestinal lumen. The host intestinal epithelium, which ensures the anatomical separation of gut microbiota from the host, plays a key role in regulating immune responses and supporting mutualistic associations between the host and gut microbiota (Schroeder and Bäckhed, 2016; Kundu et al., 2017; Levy et al., 2017). The intestinal epithelial cells are connected to each other by intercellular junctions (tight junctions, adhesion junctions, and desmosomes). These junctions work together with selective permeability, which allows the passage of nutrients across the epithelial layer and helps beneficial microorganisms to interact with the intestinal mucosal immune cells to influence their maturation and differentiation. They also protect the integrity of the epithelial layer. Mucosal barriers contain not only a single layer of epithelial cells but are also shielded by a thick mucus layer and various other physical (such as peristalsis) and biochemical (such as secretory immunoglobulin A) means (Turner, 2009). Below the epithelium is a host of immune cells, which act as a third line of defense to prevent the gut microbiota and pathogens from entering the systemic circulation. These gut immune cells, including macrophages, dendritic cells, T cells, and B cells, are housed within gut-associated lymphoid tissues, such as Peyer’s Patches or scattered throughout the lamina propria (Perez-Lopez et al., 2016; Kobayashi et al., 2019; Figure 2).

Gut microbiota and its specific molecules promote intestinal homeostasis, barrier integrity, and immune maturation by signaling to the innate and adaptive immune systems, leading to the host’s immune response and the release of protective peptides and cytokines. The result can be a protective response to commensal bacteria, an inflammatory response to pathogenic organisms, or a trigger for host cell apoptosis. For example, effects of *Escherichia coli Nissle*1917 through the Toll-like receptor (TLR) signaling pathway are necessary to inhibit intestinal inflammatory and maintain gut barrier homeostasis in inflammatory bowel disease (Damoogh et al., 2021).

Disrupted barrier function is linked to an increased risk of inflammation and immune-mediated diseases (Gensollen et al., 2016; von Mutius and Vercelli, 2019). Diabetes and obesity are well-known examples of impaired barrier function, which further increases inflammation (Nagpal et al., 2018; Thaiss et al., 2018). Another study suggested an association between arthritis and dysfunction of crucial barrier proteins, such as tight junction proteins (Tajik et al., 2020). However, to communicate with distant organs, gut microbial signals first need to be transmitted across the intestinal epithelium. These signals (active molecular) can be either structural components of the bacteria (such as LPS) or metabolites produced from the microbiota (SCFAs, bile acid) that affect distal organs either directly or by signaling through nerves or hormones from the gut. The microbe–host communication is essential to maintain vital functions in a healthy host, which enables the gut bacteria to connect to the immune and hormonal systems, to the brain (the gut–brain axis), and to the host.

## Exploring a Potential Gut–Brain–Bone Axis

The gut–brain axis refers to the network of connections that integrates neural, hormonal, and immunological signaling between the gut and the brain (Johnson and Foster, 2018). The communication system is bidirectional. Signaling from the brain *via* the autonomic nervous system and the hypothalamic–pituitary–adrenal axis influences processes associated with gastrointestinal homeostasis, such as peristalsis and transit, mucin production, immune functions, and intestinal permeability, relative gut microbial abundance, and gene expression patterns in certain gut microorganisms (Mayer, 2011; Moreira et al., 2016; Yu et al., 2017). Conversely, gut microbiota communicates with the brain *via* hundreds of metabolites, which are sensed by specialized cells in the gut, including enteroendocrine cells (EECs), enterochromaffin cells (ECCs), and primary or secondary afferent nerve endings. Sensing of bacterial metabolites by these cells results in neural signals to the brain and interactions with gut-based immune cells leading to local and systemic immune activation, or circulating metabolites enter the central nervous system by crossing the blood–brain barrier and directly affect neuroactivity (Rothhammer et al., 2016). Based on the gut–brain axis as a multidirectional interactive communication highway, these signaling processes influence multiple organs (the gut and non-gut organs, such as liver, brain, and bone).

In recent years, serotonin [5-hydroxytryptamine (5-HT)], an important and widely studied neurotransmitter, has been shown to regulate bone metabolism *via* the gut microbiota (Sjögren et al., 2012). 5-HT, a critical regulator of bone health, has two types depending on its synthesis site: brain-derived serotonin (BDS) and gut-derived serotonin (GDS). Interestingly, the two types of 5-HT fulfill distinct functions; gut-derived 5-HT has a negative effect on bone formation, whereas brain-derived 5-HT has the opposite effect (Pawlak et al., 2017). The enterochromaffin cells of the duodenum are responsible for GDS synthesis, which is partially regulated by gut microbiota (Reigstad et al., 2015). Both osteoblasts and osteoclasts synthesize 5-HT, express serotonin receptors, and regulate the uptake of 5-HT. In rats, an increased level of 5-HT induced bone loss (Bliziotes et al., 2006; Park et al., 2018). Another study showed that gut microbiota not only induced T cells and cytokines to regulate bone metabolism but also regulated the level of GDS by promoting a decrease in the rate-limiting enzyme of 5-HT biosynthesis, tryptophan hydroxylase-1, and an increase in serotonin transporter (Sjögren et al., 2012). However, recolonization of germ-free mice (GF mice) with a normal gut microbiota caused only a small change in the level of 5-HT after normalization of bone mass. It cannot be neglected that the intestinal microbiome regulates bone mass through 5-HT in the gut–brain axis. Therefore, the established links that have been made among microbes, the brain, and bones (the gut–brain–bone axis) support the feasibility of a novel approach in the treatment of bone disorders.

## Gut Microbiota and Bone Diseases: Connection and Communication

Previous studies have established associations between gut microbiota and a seemingly ever-increasing number of diseases, syndromes, and functional aberrations. Bone is an essential organ of the human body, which relies on the dynamic balance between osteoblasts and osteoclasts to maintain its normal function, and imbalance between osteoblasts and osteoclasts may lead to bone diseases that are also closely associated with gut microbiota. In the following paragraphs, we review the communication between gut microbiota and the host that contributes to the development and progression of some bone diseases and discuss how misconfigured signaling may contribute to these diseases.

### Osteoporosis

OP, the most prevalent metabolic bone disease affecting society and families, is characterized by the deterioration of bone microarchitecture and low bone mass and leads to bone fragility and even bone fractures (Estell and Rosen, 2021). With the general aging of the population, as many as 200 million people worldwide have OP, and approximately 9 million fractures occur annually (Pisani et al., 2016). This disease is likely the result of several complicating factors including nutrition, hormones, sex, heredity, and lifestyle. The most recognizable regulators of bone metabolism are estrogen, calcium, vitamin D, PTH, and inflammatory factors; however, the pathogenesis of OP remains under explored. Despite a range of effective compounds to reduce fracture risk, the treatment rates are still low among high-risk individuals (Pisani et al., 2016).

OP is most common in postmenopausal women; however, it occurs in individuals of any sex and at any age (Horowitz, 1993). Declining estrogen levels stimulate excessive osteoclast formation and bone resorption, driving rapid bone loss (Horowitz, 1993). Gut microbiota dysbiosis, such as lower diversity of gut microbiota, may result in a reduction in circulating estrogens because gut microbiota regulates estrogens through the secretion of β-glucuronidase, an enzyme that deconjugates estrogens into their active forms (Baker et al., 2017). These results can be verified in ovariectomy (OVX) mice, a model of postmenopausal OP; OVX induces bone loss through microbial-dependent trafficking of intestinal TNF^+^ T cells and Th17 cells owing to increased levels of IL-17a, TNF, and RANKL (Yu et al., 2021). However, probiotic treatment based on different species (mainly including *Lactobacillus*) prevents the bone loss induced by OVX (Chiang and Pan, 2011; Britton et al., 2014; Ohlsson et al., 2014; Li et al., 2016). For instance, *Lactobacillus paracasei* and *Lactobacillus plantarum* enhance bone volume/tissue volume, trabecular number, trabecular thickness, and cortical bone loss by reducing the expression of osteoclastogenic cytokines (TNF-α and IL-1β) and increased OPG expression in osteoporotic mice (Ohlsson et al., 2014). Moreover, *Bifidobacterium longum* also increases bone mass density in OVX rats with bone loss (Parvaneh et al., 2015). Furthermore, studies have demonstrated that supplementation with *L. reuteri* is an effective treatment for trabecular bone loss in mice with OP owing to glucocorticoid-induced or post-antibiotic-induced intestinal microbial dysbiosis and barrier dysfunction (Schepper et al., 2019, 2020).

As described above, sex steroid depletion increases the production of pro-inflammatory and pro-osteoclastogenic cytokines resulting in OP. Moreover, sex steroid deficiency augments inflammation in the gut by increasing intestinal permeability, allowing the translocation of bacteria, and increasing the number of bacterial antigens entering the epithelial mucosa (Li et al., 2016). In mice, the effect of gut microbiota in increasing these cytokines and reducing bone mass is dependent on NOD1 and NOD2 signaling, which elicits an inflammatory response (Ohlsson et al., 2017). Toll-like receptor 5 (TLR5) is the innate immune receptor for flagellin, and knockout mice for this receptor show changes in gut microbiota, which leads to an immune system deficiency. Studies have demonstrated that activation of TLR5 prompts osteoclast formation and bone loss in mice. Moreover, TLR5 knockout mice present with increasing periosteal expansion, which is normalized when there is a disruption in gut microbiota. This is consistent with the mediation role of gut microbiota (low microbial diversity; Guss et al., 2017).

Recently, studies have indicated a potential role of gut microbiota in the pathogenesis of OP. Compared to conventionally raised (CONV-R) mice, GF mice were protected against OP and the increase in bone turnover induced by sex steroid deprivation because of the lack of increase in TNF, RANKL, and IL-17 (Li et al., 2016). At the same time, the number of osteoclasts per bone surface were decreased. The frequency of the CD4^+^ T cells and CD11b^+^/Gr1^−^ osteoclast precursor cells in the bone marrow reduced, whereas bone formation was not affected (Sjögren et al., 2012). Consistent with this finding, GF mice colonized with conventional microbiota after sexual maturity had lower trabecular bone mass and significantly higher bone resorption marker CTX-I 1 month after colonization (Li et al., 2016; Yan et al., 2016). Similarly, a role of gut microbiota in the pathogenesis of OP was indicated by studies mainly on the development of lymphoid cells and production of circulating cytokines.

**Figure 3:**
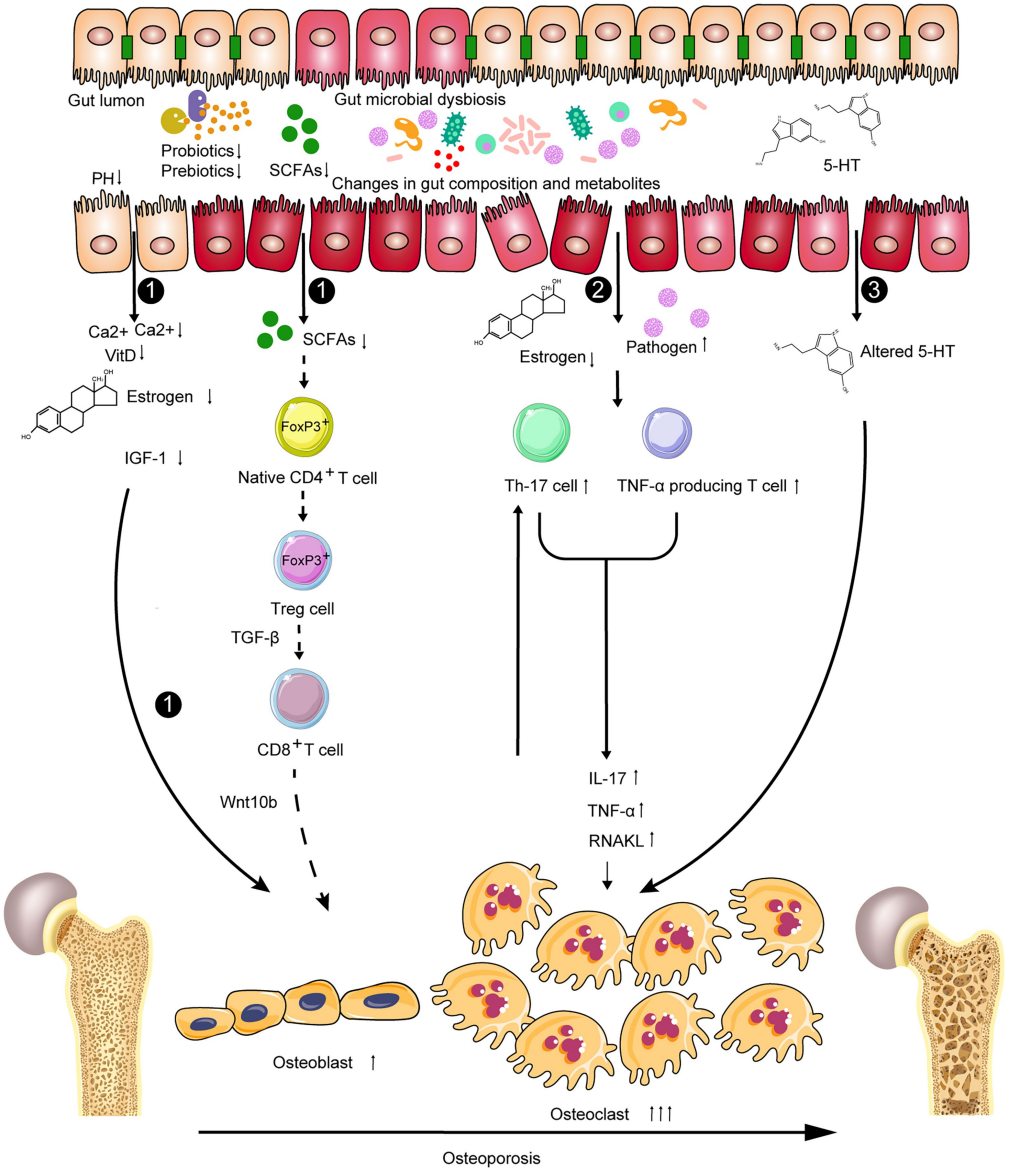
Potential gut microbiota-related mechanisms in the development of osteoporosis related to the bone remodeling balance including: 1 influencing absorption of nutrition, decreasing in estrogen biotransformation and the level of IGF-1, then regulating bone formation; 2 The GM-dependent expansion of lymphoid cells and cytokines could result in the formation and differentiation of osteoclasts thereby promoting bone destruction; and 3 changing the level of serotonin *via* the gut–brain–bone axis.

Taken together, the gut microbiota-dependent expansion of bone marrow Th17 cells and TNF-α-producing T cells, which increases their production of pro-inflammatory and pro-osteoclastogenic cytokines, such as TNF-α, IL-17, and RANKL, and decreases the secretion of the RANKL antagonist OPG (D’Amelio et al., 2008; Pacifici, 2012), may be the potential immunomodulatory mechanism in the development of OP. RANKL induces osteoclast formation, TNF potentiates RANKL activity and induces the expansion of Th17 cells, and IL-17 reduces bone formation (Cenci et al., 2000; Lam et al., 2000; Chen et al., 2011; Sugita et al., 2012; Li et al., 2015; Uluçkan et al., 2016). In addition, newly produced osteoclasts activate TNF-α-producing CD4^+^ T cells and induce the production of Treg cells (Ibáñez et al., 2016). Th17 cells, an osteoclastogenic population of CD4^+^ T cells, are defined by their ability to produce IL-17 and also secrete RANKL and TNF (Miossec et al., 2009; Basu et al., 2013), whereas Treg cells (differentiation from native CD4^+^ T cells through the intrinsic epigenetic upregulation of the transcription factor gene *FOXP3* in CD4^+^ T cells) suppress immune responses, which can induce and maintain the immune tolerance of the host, and decrease chronic inflammation *via* various mechanisms including the production of the immunosuppressive cytokines IL-10 and TGF-β (Bollrath and Powrie, 2013; Tyagi et al., 2018). As described above, the relationship between Th17 cells and Treg cells is complex; it can either inhibit or promote bone health. However, intestinal bacteria may control this balance (Figure 3).

IGF-1, an important hormone mainly produced in the liver and regulated by microbes and microbial products, plays a vital role in modulating skeletal development and postnatal growth. For example, colonization of sexually mature germ-free (GF) mice with conventional specific pathogen-free (SPF) gut microbiota increased both bone formation and resorption and increased circulating IGF-1. SCFA supplementation in antibiotic-treated mice restores serum IGF-1 and bone mass to the levels equivalent to those in non-antibiotic-treated mice. The study indicates that SCFAs produced by gut microbiota *via* the fermentation of non-digestible polysaccharides positively promote the production of serum IGF-1, and gut microbiota may provide an anabolic stimulus to the skeleton through IGF-1 (Yan et al., 2016; Shen et al., 2021).

The majority of these findings demonstrate that GF mice have increased bone mass. In contrast to these observations, a study by Schwarzer and colleagues demonstrated that femur length is longer, and trabecular and cortical bone mass is significantly higher in CONV-R mice than in GF mice, with no difference in the bone mineral density (Schwarzer et al., 2016). Several studies on mice treated with antibiotics to alter the composition of gut microbiota present different conclusions regarding the effect on bone density (Cho et al., 2012; Cox et al., 2014). To sum up, these discrepancies may be explained by the differences in the bone mass phenotypes of GF mice and bone density measurement and antibiotic treatment protocols used (Cho et al., 2012; Sjögren et al., 2012; Cox et al., 2014; Li et al., 2016; Schwarzer et al., 2016; Yan et al., 2016).

However, data on the association between the human gut microbiota and OP is very limited. Recently, it was shown that gut microbiota structure and diversity, especially the abundance of *Dialister* and *Faecalibacterium*, are significantly enriched in patients with OP compared with those in healthy controls. However, the findings link the differences in gut microbiota components neither with inflammation and immune system nor with bone turnover (Xu et al., 2020). Moreover, it has been shown that probiotics also affect bone health in humans. Intervention with *Lactobacillus casei* Shirota in elderly men and women improved distal radius fracture healing (Lei et al., 2016), and another study showed that in healthy postmenopausal Japanese women, the total hip bone mineral density was increased in the group treated by the probiotic *Bacillus subtilis* C-3102 over 6 months compared with the control group treated by the placebo (Takimoto et al., 2018; Table 1).

**Table 1:** Summary of studies evaluating the effects of probiotics on bone outcomes in human.

Probiotics	Study subjects	Study design	Effects	References
*Lactobacillus casei shirota*	Elderly patients with distal radius fracture, *n* = 381	RCT, D = 6 months	DASH score, pain, CRPS score, wrist flexion and grip strength of patients receiving probiotics exhibited a faster rate and improve distal radius fracture healing vs. the control group.	Lei et al., 2016
*Bacillus subtilis C-3102*	Postmenopausal women, *n* = 53	RCT, D = 24 weeks	uNTx and TRACP-5b decreased in the probiotic group at 12 weeks vs. control group, the total hip BMD increased in the probiotic group vs. control group.	Takimoto et al., 2018
*Lactobacillus casei Shirota*	Older individuals with knee OA, *n* = 433	RCT, D = 6 months	WOMAC and VAS scores improved in the probiotics group vs. control group. Serum levels of hs-CRP were lower in patients receiving probiotics than control, and probiotic improved treatment knee OA.	Lei et al., 2017
*Streptococcus thermophilus* (TCI633)	Participants with knee OA, *n* = 67	RCT, D = 12 weeks	sCTX-II, sCRP, and WOMAC scores improved and the progression and development of knee OA retard in the patients receiving probiotics vs. control group.	Lyu et al., 2020
*Lactobacillus casei* 01	Patients with RA, *n* = 46	RCT, D = 8 weeks	TNF-α, IL-6, and IL-12 decreased, IL-10 was increased in the probiotic group vs. control group, probiotics supplementation improved the inflammatory status of patients with RA.	Paul et al., 2021

RCT, randomized, placebo-controlled, double-blind clinical trial; D, duration; DASH score, disabilities of the arm, shoulder and hand score; CRPS score, complex regional pain syndrome score; uNTx, urinary type I collagen cross-linked N-telopeptide; TRACP-5b, tartrate-resistant acid phosphatase isoform 5b; BMD, bone mineral density; OA, osteoarthritis; WOMAC, Western Ontario and McMaster Universities Osteoarthritis Index; and VAS scores, visual analog scale scores. hs-CRP, high sensitivity C-reactive protein; sCTX-II, serum collagen type II C-telopeptide; sCRP, serum C-reactive protein; RA, rheumatoid arthritis; TNF-*α*, tumor necrosis factor-*α*; IL-6, interleukin-6; IL-12, interleukin-12; and IL-10, interleukin-10.

### Osteoarthritis

OA, the most common type of arthritis, is a degenerative disease with low-grade inflammation, a leading cause of joint pain and disability worldwide, especially among the elderly (2018). Pathological changes in OA affect all joint tissues, leading to degradation of cartilage and bone, abnormal bone formation (osteophytes), and inflammation of the synovial membrane (synovitis). Previous studies have shown that obesity and metabolic syndrome are well-known risk factors for OA through overweight load on joint and low-grade systemic inflammation, and gut microbiota is implicated in its pathogenesis (Yoshimura et al., 2012; Johnson and Hunter, 2014; Huang and Kraus, 2016). In general, gut microbiota alleviates obesity through various mechanisms, which would have a positive influence on modifying the risk of OA. Accordingly, some animal studies with ingenious designs demonstrated that mice following a long-term high-fat diet or high-sucrose diet are prone to develop obesity-mediated knee OA (Griffin et al., 2012; Collins et al., 2015). However, this risk and the symptoms are reduced by intervention with *L. casei* or the prebiotic oligofructose (So et al., 2011; Schott et al., 2018). Moreover, other studies recently demonstrated that changes in the abundance of *Fusobacterium*, *Faecalibacterium*, and *Ruminococcus* play a key role in exacerbating OA in mice. In addition, studies show that high levels of lipopolysaccharide (LPS) in the serum and synovial fluid are associated with knee OA severity, macrophage-associated inflammation, and worsening of OA pathology (Huang and Kraus, 2016; Huang et al., 2016). Moreover, a large population study in adults recently validated that the abundance of *Streptococcus* species is associated with increased knee pain driven by local inflammation in the knee joint (Boer et al., 2019). Notably, randomized, double-blind, placebo-controlled clinical trials in patients with knee OA have also shown that probiotics *L. casei* Shirota and *Streptococcus thermophilus* (TCI633) have a positive effect of improvement in knee OA (Lei et al., 2017; Lyu et al., 2020; Table 1).

### Rheumatoid Arthritis

RA is a systemic chronic inflammatory disease mediated by immune system that leads to progressive joint destruction and impairs the quality of life. To date, many studies have focused on RA to study the role microbiota in autoimmunity. Modified gut microbiota structure has been observed in RA in both animal and human studies (Lyu et al., 2020). In RA cases, *Prevotella* and various *Lactobacillus* species have been shown to be more abundant at the species level (Liu et al., 2013). Therefore, the increased abundance of *Prevotella* and imbalance in the gastrointestinal microbiota are potential sources of the initiation or progression of RA. However, the mechanism of action of *Prevotella*-induced RA remains unknown. Probiotics containing *Lactobacillus* spp. (mainly *L. casei* and *Lactobacillus acidophilus*) are commonly used as alleviating agents or food supplements to manage RA and maintain general health (Vaghef-Mehrabany et al., 2014; Paul et al., 2021; Table 1).

### Bone Cancer

Recently, emerging scientific advances have significantly contributed to the understanding of the potential associations between gut microbiota and the initiation, progression, and prognosis of multiple cancer types (Sepich-Poore et al., 2021). The International Agency for Research on Cancer has designated specific pathogens as procarcinogens and carcinogens in gastrointestinal and non-gastrointestinal cancers in humans (de Martel et al., 2020). Among them, *Helicobacter pylori*, which colonizes the gastric mucosa, accounts for approximately 50% of individuals worldwide; it is identified as the main cause of chronic gastric inflammation (Suerbaum and Michetti, 2002), and its role in the etiology of human gastric cancer has also been identified (Alfarouk et al., 2019). The potential mechanism through which *H. pylori* mediates the carcinogenesis of gastric cancer is associated with the presence of urease, Lewis antigens, cytotoxin-associated gene A, vacuolating cytotoxin, and BabA2 and induces chronic inflammation and promotes carcinogenesis (Alfarouk et al., 2019). The gram-negative anaerobic commensal *Bacteroides fragilis* has been identified to be potentially involved in colorectal tumorigenesis (Allen et al., 2019; Maiuri et al., 2019; DeStefano Shields et al., 2021), and there is emerging evidence for the involvement of *Acidovorax* (phylum Proteobacteria) in the development of lung cancer (Greathouse et al., 2018). Advances in 16S rRNA gene sequencing technology have opened up a new frontier in human gut microbiota analysis during cancer onset or progression. Metabolomics or metatranscriptomics have further advanced microbiome analyses, allowing researchers to characterize both the microbiome and its function in the development of human cancers. This knowledge of the gut microbiome in a multitude of human cancers offers an insight into the development of microbial-based diagnosis and treatments with the aim of enhancing patient care and outcomes.

Bone cancer, especially osteosarcoma, usually occurs in children and adolescents. Although the clinical treatment (including surgical techniques, chemotherapy, and radiotherapy) has advanced significantly in the last few decades, the outcome is still poor in patients with metastatic disease at present or in relapse, resulting in a high mortality rate. Thus, bone cancer treatment remains a major challenge worldwide. To date, the association between gut microbiota and bone tumor has not been reported. Herein, we focus on studying the characteristics of gut microbiota in patients with bone tumor and how specific bacteria and its structure affect tumor development and progression. Our study will explore a field to combine bone tumor and gut microbiota and may provide feasible prevention and therapy for bone cancer-related disease. It is of great significance of improve the prognosis and survival rate of patients.

## Conclusion and Future Perspectives

Over the past decades, experiments using different approaches and studies in humans have provided remarkable novel insights into the complex interplay between gut microbiota and bone diseases. We analyzed recent studies that address the association between gut microbiota and certain skeletal disorders and propose potential mechanisms. Thus, gut microbiota has emerged as an important regulator of bone homeostasis. Nevertheless, current evidence is limited and often indirect. For example, an understanding of the interaction between gut microbiota and OP pathology and treatment (probiotics, prebiotics, fecal microbiota transplantation) is mostly derived from animal model studies that explored the contribution of gut microbiota in bone metabolic pathways (related to calcium and hormones, for example) rather than the effect of specific gut microbiota and its characteristics in these processes. However, the interactions have only rarely been assessed in humans. Therefore, well-controlled clinical trials are needed to explore how differences in microbiota composition affect different bone disorders including bone cancer. Further understanding of these aspects may aid the development of tools that take into consideration patients’ gut microbiomes and guide advances in targeted therapeutics based on gut microbiota, which may have the potential to improve the efficacy of these therapeutics.

## Author Contributions

YC and ZR designed the study. YC, XW, CZ, and CL retrieve references and analyze data. YC wrote the manuscript. ZR revised the manuscript. All authors contributed to the article and approved the submitted version.

## Funding

This study was sponsored by grants from National Key Research and Development Program of China (2018YFC2000501), National Natural Science Foundation of China (U2004121), and China Postdoctoral Science Foundation (2020T130609).

## Conflict of Interest

The authors declare that the research was conducted in the absence of any commercial or financial relationships that could be construed as a potential conflict of interest.

## Publisher’s Note

All claims expressed in this article are solely those of the authors and do not necessarily represent those of their affiliated organizations, or those of the publisher, the editors and the reviewers. Any product that may be evaluated in this article, or claim that may be made by its manufacturer, is not guaranteed or endorsed by the publisher.

## Abbreviations

OP: Osteoporosis
OA: Osteoarthritis
RA: Rheumatoid arthritis
PTH: Parathyroid hormone
SCFAs: Short-chain fatty acids
HDACs: Histone deacetylases
OPG: Osteoprotegerin
Treg cells: T Regulatory cells
IGF-1: Insulin-like growth factor 1
TLR: Toll-like receptor
5-HT: 5-Hydroxytryptamine
GDS: Gut-derived serotonin
GF mice: Germ-free mice
OVX: Ovariectomy
CONV-R mice: Conventionally raised mice
SPF: Specific pathogen-free
